# The transnasal endoscopic approach for resection of clival tumors: a single-center experience

**DOI:** 10.1038/s41598-023-30216-8

**Published:** 2023-02-21

**Authors:** Vicki M. Butenschoen, Philipp Krauss, Denise Bernhardt, Chiara Negwer, Stefanie Combs, Bernhard Meyer, Jens Gempt

**Affiliations:** 1 Department of Neurosurgery, School of Medicine, Klinikum rechts der Isar, Technical University of Munich, Ismaningerstr. 22, 81675 Munich, Germany; 2 Department of Radiation Oncology, School of Medicine, Klinikum rechts der Isar, Technical University of Munich, Ismaningerstr. 22, 81675 Munich, Germany; 3 Department of Neurosurgery, Faculty of Medicine, University of Augsburg, Stenglinstraße 2, 86156 Augsburg, Germany; 4 Department of Radiation Sciences (DRS), Institute of Radiation Medicine (IRM), Helmholtz Zentrum München (HMGU), Ingolstädter Landstraße Ingolstädter Landstraße 1, 85764 Oberschleißheim, Germany; 5 Deutsches Konsortium für Translationale Krebsforschung (DKTK), Partner Sites Munich, Munich, Germany

**Keywords:** CNS cancer, Outcomes research, Neurological disorders

## Abstract

Clival tumors present challenging entities regarding their treatment options. Due to their proximity to critical neurovascular structures, the operative goal of gross total tumor resection is rendered more difficult by a high risk of neurological deficits. Retrospective cohort study of patients treated for clival neoplasms through a transnasal endoscopic approach between 2009 and 2020. Assessment of preoperative clinical status, length of operation, number of approaches, pre- and postoperative radiotherapy, and the clinical outcome. Presentation and clinical correlation with our new classification. In total, 59 transnasal endoscopic operations were performed on 42 patients over 12 years. Most lesions were clival chordomas; 63% of the lesions did not reach the brainstem. Cranial nerve impairment was present in 67% of the patients, and 75% of the patients with cranial nerve palsy improved after surgical treatment. Interrater reliability for our proposed tumor extension classification showed a substantial agreement (Cohen’s κ = 0.766). The transnasal approach was sufficient to achieve a complete tumor resection in 74% of the patients. Clival tumors exhibit heterogeneous characteristics. Depending on clival tumor extension, the transnasal endoscopic approach can present a safe surgical technique for upper and middle clival tumor resection, with a low risk of perioperative complications and a high rate of postoperative improvement.

## Introduction

Midline tumors of the clivus located at the posterior cranial fossa may cause cranial nerve impairment^1^ or brainstem compression. Due to their anatomical location and close proximity to critical neurovascular structures such as caudal cranial nerves and basilar and carotid arteries^2^, pathologies of the clivus are generally difficult to treat^3,4^, and gross total resection (GTR) may require several attempts and approaches^5–7^. Despite their challenging nature, current evidence on clival pathologies lacks specific classifications with prognostic value for the extent of resection (EOR) and progression-free survival. Several authors have offered grading depending on anatomical location^8,9^, growth pattern^10^ and histopathological features^11^.

The aim of this study was to assess the clinical outcomes of patients undergoing transnasal endoscopic resection of clival tumors, and to present a new tumor extent classification to estimate the complication rate and probability of a single-staged approach.

## Methods

### Study cohort

We performed a consecutive case study of all patients treated endoscopically for tumors of the clivus, reviewing all patients who received operations via a transnasal endoscopic approach in our neurosurgical department between January 2009 and January 2020. We excluded patients suffering from skull base osteomyelitis or degenerative pathologies such as basilar invagination due to their distinct clinical outcome.

Pre- and postoperative data (with description of the surgical technique, EOR, and pre- and postoperative imaging) were retrieved from our records. We reviewed the preoperative goal of the operation (GTR, subtotal resection [STR], tumor debulking, and biopsy); clinical information included occurrence and side of cranial nerve palsy and duration of symptoms. The clinical status before and after operations and in follow-up was assessed according to the Karnofsky Performance Status Scale (KPSS).

If available, preoperative diagnostic imaging included pre- and postoperative computed tomography (CT) and magnetic resonance imaging (MRI) of the craniovertebral junction.

### Classification

**Figure 1:**
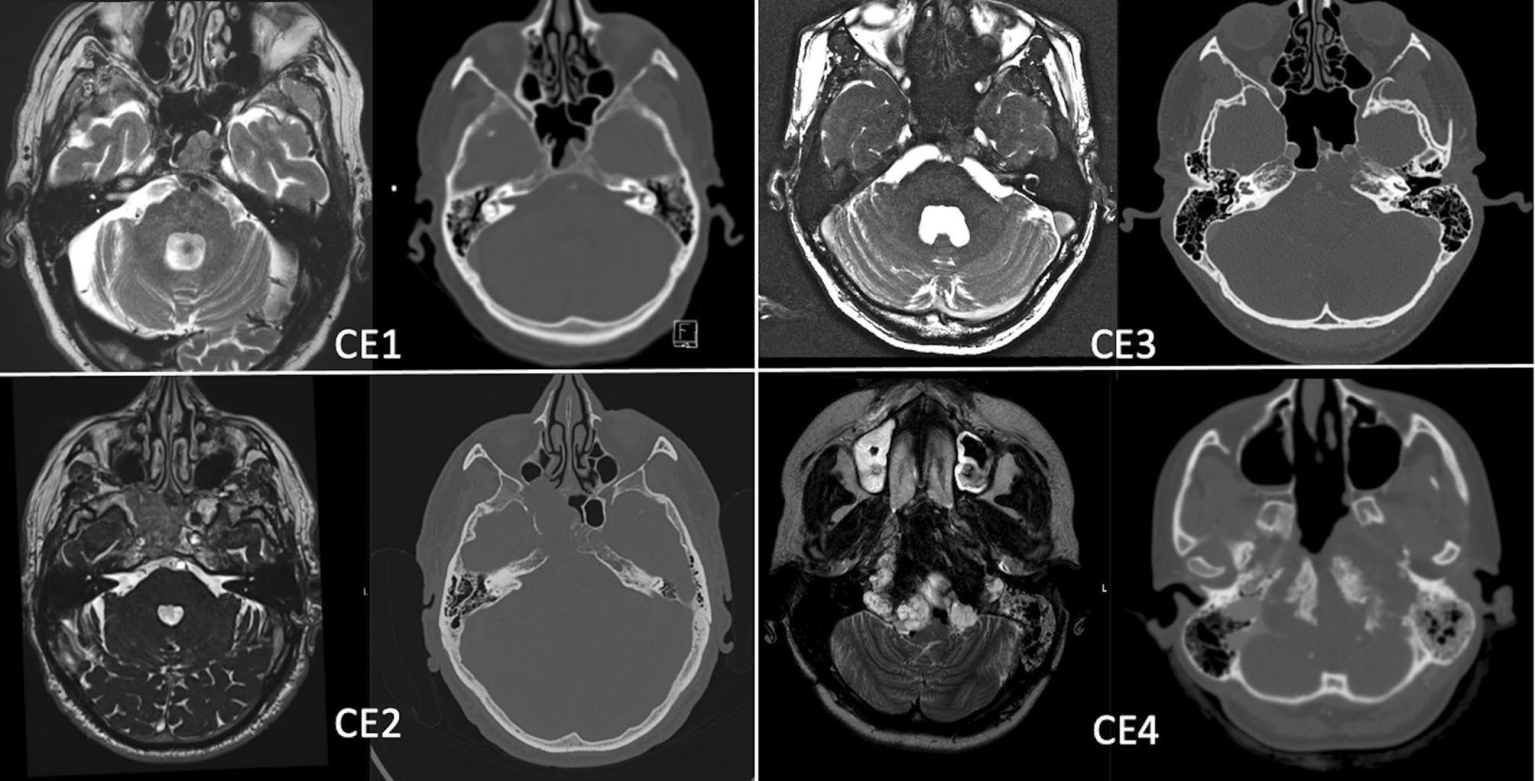
Clival extent classification (CE) with various extent categories: CE1: Intraosseous lesion, CE2: Destruction of the osseous clival cortical bone, CE3: Brainstem reached without compression or infiltration, CE4: Compression/infiltration of the brainstem.

We classified tumor extension in 4 distinct subgroups: clival extension (CE) 1–4 depending on the preoperative CT and MR imaging. The classification was evaluated by 2 individual reviewers (Fig. 1):

- CE1: Intraosseous lesion
- CE2: Destruction of the osseous clival cortical bone
- CE3: Brainstem reached without compression or infiltration
- CE4: Compression/infiltration of the brainstem

The aim of this simple classification was to assess and quantify the risk and occurrence of complications based on imaging without histopathological diagnosis before the operation.

### Study design

We conducted a retrospective single-center analysis in a tertiary neurosurgical center. We assessed relevant details leading to the indication of the surgical intervention, intraoperative findings and procedures, and postoperative radiographic and clinical outcomes, depending on classification based on tumor extension as defined by the preoperative MRI and CT scans.

### Ethics

The presented study meets the ethical standards outlined in the Declaration of Helsinki. All the study protocol were approved by the ethics committee of Technical University Munich, registration number 231/20 S (Prof. Schmidt, Technical University Munich). Due to the retrospective nature of the study, patient consent was not required and was therefore waived by the local ethics committee (Prof. Schmidt, Technical University Munich).

Written informed consent was obtained from our patient presented in the case presentation.

### Statistics

We performed statistical analyses using SPSS Statistics 26 (IBM, Chicago, IL). Binomial dichotomized data were compared using Fisher’s exact test, and categorical data were compared using the χ^2^ test. Likelihood ratio (LR) was used to assess changes in odds.

Median or mean values were compared using a student’s *t* test when appropriate. The association between potential factors and transient and permanent postoperative impairment (using follow-up data or discharge data for those with a missing follow-up) was analyzed using ANOVA for variance testing and linear regression modeling. The following factors were assumed as potentially predictive: symptom duration, length of operation (LOO), EOR, CE1-4 classification, preoperative treatment such as previous radiation therapy, tumor malignancy, and age. Interrater reliability (IRR) was assessed with the Cohen’s κ coefficient.

*p* values less than 0.05 were considered statistically significant.

## Results

### Patient population

In total, 59 transnasal endoscopic operations were performed in 42 patients over 12 years. All 42 patients had neoplastic lesions. Median age was 53 years (IQ range 29–69 years), and 25/42 (59.5%) of the patients were females. Median symptom duration was 39 days (IQ range 14–154 days).

Most cases (35/42, 83.3%) presented with clinical symptoms related to the clival lesion, such as cranial nerve palsy (28/42, 66.7%, mostly the abducens nerve [14/28, 50%]), followed by the glossopharyngeal nerve (9/28, 32%) and oculomotor nerve (3/28, 10.7%). In 19/42 patients, lesions were diagnosed due to headache symptoms (45.2%) (Table 1).

**Table 1:** Patient demographics including median age, median symptom duration, percentage of patients suffering from preoperative cranial nerve palsy and headache, and median Karnofsky Performance Status Scale (KPSS).

		Range
Age (years)	Median 53	IQ 29–69
Symptom duration (days)	Median 39	IQ 14–154
Cranial nerve palsy	66.7%	Yes/No
Headache	45.2%	Yes/No
KPSS	Median 90%	20–100%

Most patients suffered from lesions of the middle clivus (26/42, 61.9%), followed by tumors including all parts of the clivus (7/42, 16.7%), the upper clivus (5/42, 11.9%) and the lower clivus (4/42, 9.5%). In 7/42 (16.7%) patients, tumors were identified incidentally during regular CT staging for tumor follow-up. In 3 patients (7.1%), the initial endoscopic operation was performed after a previous radiation therapy.

Median preoperative KPSS was 90% (range 20–100%). Preoperative MRI, CT scan, and navigation were available for all patients.

### Tumor histology and classification

Slow-growing tumors were diagnosed in 26/42 (61.9%) cases: chordoma 16/42 (38.1%), chondrosarcoma G1 4/42 (9.5%), hamartoma 1/42 (2.4%), Rathke’s cleft cyst 2/42 (4.8%), Langerhans-cell histiocytosis 1/42 (2.4%), hemangioma 1/42 (2.4%), or epidermoid 1/42 (2.4%).

In 16/42 (38.1%) patients, the histopathological analysis revealed a malignant tumor: metastasis 8/42 (19%), adenoid cystic carcinoma 2/42 (4.8%), plasmocytoma 1/42 (2.4%), rhabdomyosarcoma 1/42 (2.4%), chondrosarcoma G3 1/42 (2.4%), aggressive dedifferentiated chordoma 1/42 (2.4%), neuroblastoma 1/42 (2.4%), and one neuroendocrine tumor (2.4%).

Using the CE classification described above, most patients (20/42, 47.6%) had lesions with erosion of the cortical bone without contact with the brainstem (classified as CE2). Intraosseous lesions (CE1) were identified in 6/42 (14%), compression of the brainstem (CE4) in 10/42 (23.3%) and extent to the brainstem without compression (CE3) in 6/42 patients (14%) (Table 2).

**Table 2:** Histopathological and surgical data, as well as subgroups according to the implemented CE classification based on extent of the lesions: CE1: Intraosseous lesion, CE2: Destruction of the osseous clival cortical bone, CE3: Brainstem reached without compression or infiltration, CE4: Compression/infiltration of the brainstem.

	N (/total)	*%*
Tumor lesion	42/42	100
Benign tumor	26/42	61.9
Malign tumor	16/42	38.1
CE classification		
CE1	6/42	14.3
CE2	20/42	47.6
CE3	6/42	14.3
CE4	10/42	23.8
Mean surgery duration	153 min	Range 20–485 min
GTR	21/59	35.6
STR	26/59	44.1
Tumor debulking	5/59	8.5
Biopsy	7/59	11.9

### Surgical approach

The mean duration of operations was 153 min (SD 97 min, range 20–485 min). All operations were performed with 18-cm endoscopes of 0° and 30° (Storz, Tuttlingen, Germany). The 4-handed technique via both nostrils was used in accordance with the technique described in previous publications from our study group^12^.

Preoperative MR and CT imaging as well as intraoperative navigation were used in all cases (BrainLAB, Munich, Germany). Intraoperative leakage of cerebrospinal fluid (CSF) was observed during 19/59 (32.2%) operations, and dural reconstruction was performed in 24/59 (40.7%) operations (autologous fascia lata used in 13/59 [21.3%] cases and nasal septum flap in 13/59 [21.3%] cases). Intraoperative CT imaging was conducted in 5/59 (8.5%) cases.

**Figure 2:**
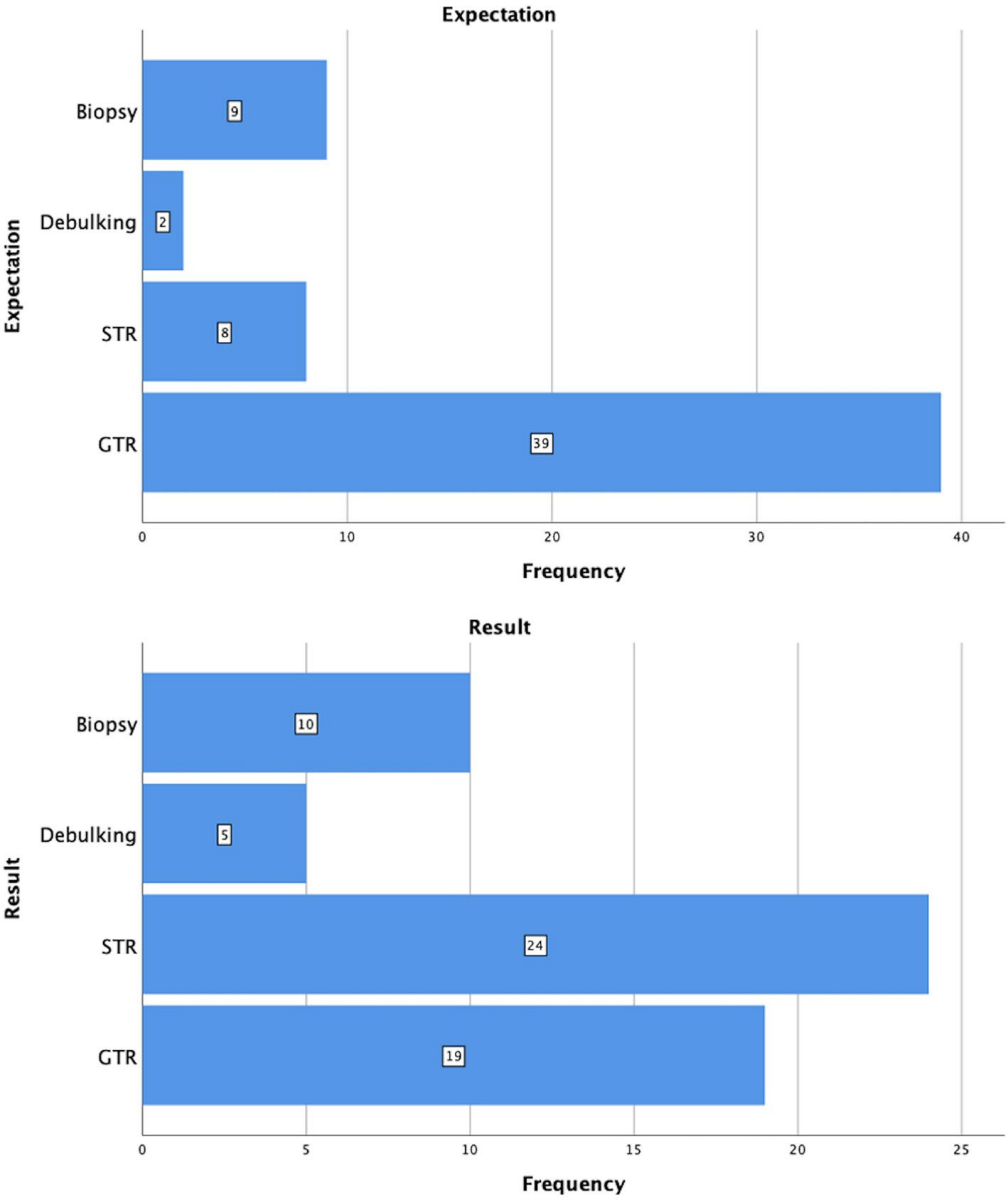
Expectations vs. results in transnasal endoscopic resection of clival tumors.

In 43/59 (72.9%) of operations, the preoperative goal was GTR of the lesion. In 6/59 (10.2%), the initial indication for operation was a diagnostic biopsy. STR or tumor debulking were the goals in 8/59 (13.6%) and 2/59 (3.4%) cases, respectively (Fig. 2).

Overall, GTR was achieved in 21/59 (35.6%) operations, STR in 26/59 (44.1%), tumor debulking in 5/59 (8.5%), and only a biopsy in 7/59 (11.9%) cases (Table 2).

In 18/42 (43%) patients, 2 or more operations were needed to complete a GTR due to residual tumor on the postoperative MRI (15/18 patients, 83%) or tumor recurrence during follow-up (3/18 patients 17%). The relative risk for multiple approaches was higher in patients suffering from lesions of the lower clivus (75%) compared to patients suffering from upper or middle clivus pathologies, but the association failed to reach statistical significance (OR 7.3, *p* = 0.063). Patients undergoing a second surgical tumor resection during the initial hospital stay underwent a second transnasal endoscopic approach (7/15, 47%) or a different transcranial approach (6 patients: lateral suboccipital in 3 patients, subtemporal approach in 2 patients, and antero-cervical in one patient). Two patients underwent a dorsal craniocervical fixation.

### Prognostic factors and CE classification

The implemented and presented classification of CE1 to CE4 was evaluated by 2 independent reviewers assessing the preoperative MRI and CT scans of all patients, showing a substantial IRR of Cohen’s κ: 0.766.

The CE classification significantly correlated with the postoperative KPSS (*p* = 0.045), necessity of staged approaches for tumor resection (*p* = 0.005, Table 3, odds ratio [OR] for CE4 tumors = 6.9), need for dural reconstruction (*p* = 0.017), and occurrence of complications (*p* = 0.009). Preoperative radiotherapy showed a trend of increased risk for perioperative complications but failed to reach statistical significance (*p* = 0.098, OR = 6.7).

**Table 3:** LOH (length of hospital stay), percentage of patients undergoing 2 or more operations for tumor resection, LOO (length of operation), and postoperative Karnofsky Performance Status Scale (KPSS).

	CE1	CE2	CE3	CE4	*p*
LOH (mean, days)	10.5	10.3	13.2	20	0.323
2 or more surgical approaches (%)	0	28.6	33.3	70	0.005**
LOS (mean, min)	82	132	138	223	0.022*
KPSS (median)	90	90	90	80	0.045*
Complications (%)	0	11.8	50	50	0.009**

CE1: Intraosseous lesion, CE2: Destruction of the osseous clival cortical bone, CE3: Brainstem reached without compression or infiltration, CE4: Compression/infiltration of the brainstem, *p < 0.05, **p < 0.01.

### Postoperative outcome and adverse events

Adverse events occurred in 22% (13/59) postoperative courses. CSF leakage requiring surgical revision occurred in only 2/59 (3.4%). Adverse events that did not require surgical revision included disorders of sodium balance after 3/59 operations (5.1%), hypocortisolism with transient hydrocortisone substitution in one case (1.7%), a urinary tract infection in 1/59 (1.7%), respiratory complications in 2 cases (3.4%), and meningitis in one case (1.7%). Thirty-day mortality was 0%.

The median length of hospital stay was 9 days (range 3–80 days, IQ range 5–19 days). Median postoperative KPSS was 90% (range 20–100%).

Data on recommended adjuvant therapy was available for all patients and recommended in 33/42 (79%) of them: 16/42 (38.1%) underwent postoperative photon radiotherapy (median total dose 54 Gray [Gy], range 36–70.4 Gy), and 17/42 (40.5%) patients were recommended proton beam radiotherapy of the clival area (maximum total dose 74 Gy). In total, 9/42 (21.4%) patients underwent follow-up imaging without further adjuvant treatment.

Follow-up data was available for 34/42 (81%) of patients after a median of 37 months (IQ 6–60 months). At follow-up, 12/16 (75%) patients with previous preoperative cranial nerve palsy had recovered completely, and 18/34 (52.9%) of the patients remained stable at follow-up (15 patients remained stable without exhibiting cranial nerve palsies prior to the surgical treatment, leaving only 3 patients with persistent cranial nerve palsy after surgical treatment). Tumor reoccurrence (3/4) or postoperative worsening of previous neurological status (1/4) accounted for the 4/34 (11.7%) patients who had deteriorated at follow-up.

### Case presentation

**Figure 3:**
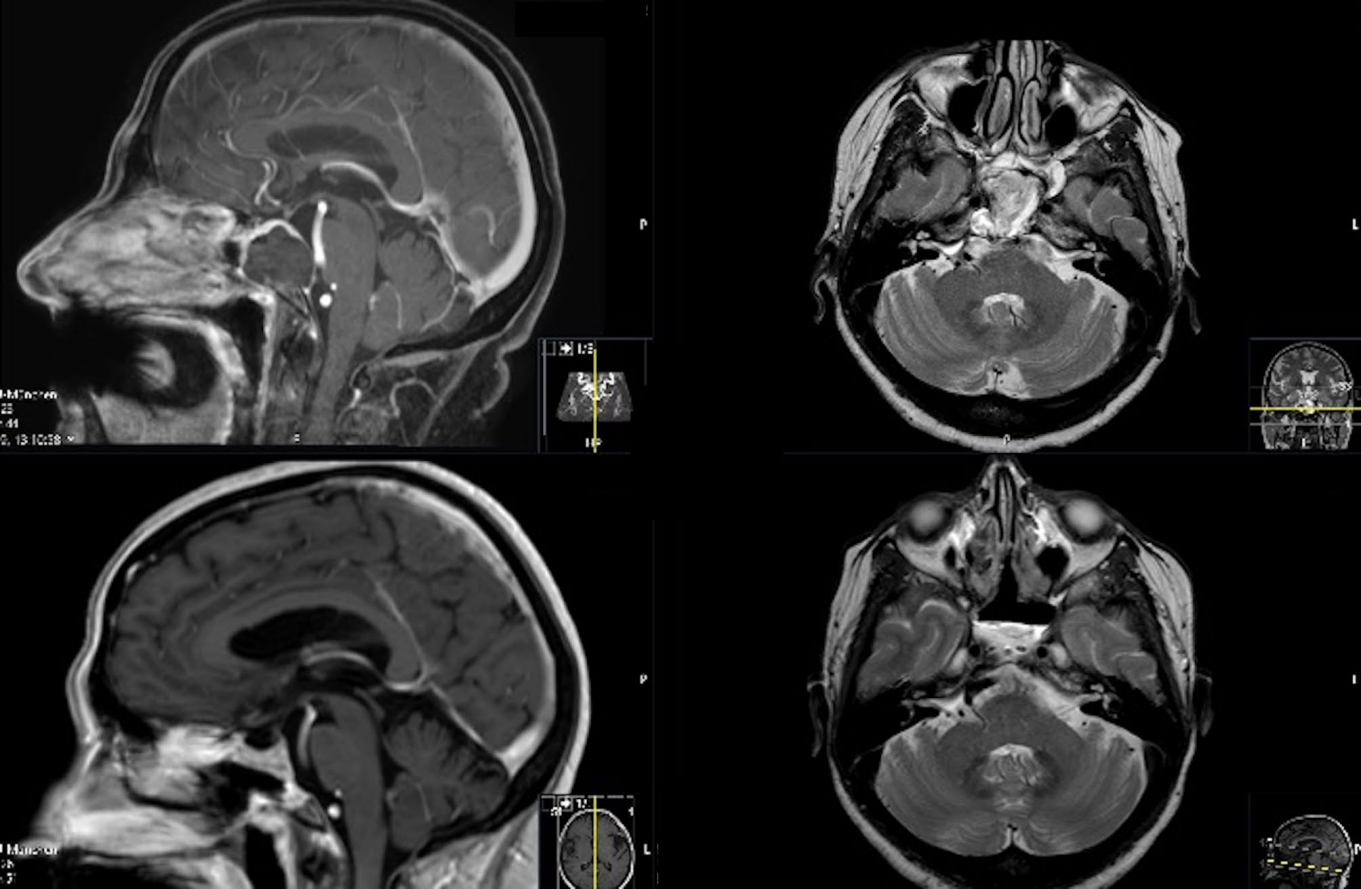
Preoperative T1 with contrast enhancement and T2-weighted MRI scan of a 60-year-old female patient suffering from a clival chordoma (upper images), with postoperative MRI revealing a gross total tumor resection after the transnasal approach.

A 60-year-old female patient presented in the outpatient department with paresis of the abducens nerve on the right side. The initial CT and MRI scans revealed an osteodestructive mass of the clivus (Fig. 3). A transnasal transsphenoidal endoscopy was performed with a duration of 137 min; GTR was confirmed by the postoperative MRI (Fig. 3). Histopathological analysis revealed a clival chordoma with a proliferation index of 6%. The patient recovered from her cranial nerve paresis and underwent radiotherapy with a total dosage of 54 Gy over 6 weeks. After a follow-up of 10 years, she remains tumor-free.

## Discussion

Clival tumors are rare and comprise various types of tumor entities with distinct histopathological and anatomical features. Depending on the type of neoplasm and the extent of the lesion, patients may present with cranial nerve palsies or symptoms of intracranial hypertension.

Preoperative planning and patient advising should not only include the possible entities, but also the prospect of multiple operations and possible neurological outcomes after operative treatment. Up to now, anatomical classifications to estimate the extent of clival lesions have been sparse, and none of them focus on the relation of the tumorous lesion to the brainstem or intra/transclival extension (involvement of the corticalis). We classified clival tumor extension with high IRR, prognostic value for neurological outcome (KPSS), and the occurrence of multiple approaches (reoperation, expectation, and EOR) in patients undergoing a transnasal endoscopic approach.

### Surgical approaches

In our study, we included patients undergoing a transnasal endoscopic approach for clival pathologies. Our aim was to include all patients with transnasal endoscopic operative treatment of clival tumors to describe the outcome of this specific technique, as patients usually undergo transnasal treatment for minimally invasive biopsy or tumor resection. The optimal approach for clival tumor resection remains a matter of debate. The advantages of the transnasal endoscopic approach remain the limited invasiveness, wide exposure of the clivus^13,14^ and acceptable morbidity compared to more open lateral approaches, such as the subtemporal^15^, far lateral^16^ or transpetrosal approach^17^. In our patient cohort, the transnasal endoscopic approach provided excellent resection results with a low complication rate. Further operative treatment from a different transcranial approach was necessary in only 6/42 patients (14%).

### Prognostic value of the classification

In our study, we aimed to provide a simple preoperative grading method to advise patients undergoing an initial transnasal endoscopic approach for suspected clival tumor lesions. Our grading system was not applied to patients undergoing other surgical approaches and did not require the histopathological diagnosis of a chordoma.

Some classifications have been proposed for grading and scoring specific tumor entities of the skull base, such as the Sekhar Grading System for Cranial Chordomas^18^. This classification included 4 criteria (size, site, and vascular and dural invasion) and was applied to histologically confirmed chordomas. Compared to our study, other researchers included patients who mostly underwent an open microscopic approach (far lateral or extended subfrontal) and required the histopathological confirmation of a diagnosed chordoma.

### Neurological outcome and surgical adverse events

In our cohort population, two-thirds of the patients presented with cranial nerve palsies, congruent with current literature describing mostly deficits due to compression of the abducens nerve in Dorello’s canal (45% in chordomas, 100% in clival metastases)^2,19^. The recovery rate was satisfying, with 75% of the patients recovering from preoperative cranial nerve palsy after operation. The transnasal endoscopic approach proved to be a safe approach with a low complication rate (CSF leakage 3.4%, compared to published literature with a range of 2.5–25% CSF leakage rates)^3^.

### Limitations

Due to the limited number of cases included and the monocentric and retrospective nature of our study, the prognostic power and conclusions may be limited.

We included various tumor pathologies, as the classification is intended to be used prior to histopathological diagnosis, but the postoperative outcome significantly depends on the tumor entity and need for postoperative treatment. To estimate the real potential benefits or pitfalls of the proposed classification, it should be verified and applied to a higher number of patients in a possible multicenter study.

While the benefits of the transnasal endoscopic approach have been presented above, it has some anatomical limitations regarding the lower clivus^20,21^. Especially for lesions reaching to the lower clivus and craniovertebral junction, GTR may be limited due to a reduced exposure.

We found satisfying results applying the endoscopic transnasal approach regarding the EOR, low complication rate, and encouraging clinical outcomes. In our department, the endoscopic approach is mainly performed by 2 senior neurosurgeons with many years of experience with endoscopic skull-base operation, which may explain the low rates of CSF leaks and overall complications^22^. We did not investigate the learning curves in our department but feel that the benefits of endoscopic surgical approaches may surpass the open approaches when performed on a regular basis.

## Conclusion

The transnasal endoscopic approach remains a safe approach with a wide exposure for upper and middle clival tumors and satisfying extent of tumor resection results. More than half of the patients presenting with preoperative cranial nerve palsies recovered completely after transnasal endoscopic operations, and the overall complication rate remained low.

## Data Availability

The datasets used and/or analyzed during the current study available from the corresponding author on reasonable request.
